# New Insights into the Pleiotropic Actions of Dipeptidyl Peptidase-4 Inhibitors Beyond Glycaemic Control

**DOI:** 10.17925/EE.2024.20.2.5

**Published:** 2024-09-06

**Authors:** Safwat A Mangoura, Marwa A Ahmed, Andrew Z Zaka

**Affiliations:** 1. Department of Pharmacology and Toxicology, Faculty of Pharmacy, Badr University in Cairo (BUC), Badr, Cairo, Egypt; 2. Department of Medical Pharmacology, Faculty of Medicine, Assiut University, Assiut, Egypt

**Keywords:** Antioxidants, coronavirus, diabetes mellitus, diabetic nephropathies, dipeptidyl peptidase-4, dipeptidyl peptidase-4 inhibitors, glucagon-like peptide-1, pleiotropic actions

## Abstract

Dipeptidyl peptidase-4 (DPP-4) is a multifunctional serine ectopeptidase that cleaves and modifies a plethora of substrates, including regulatory peptides, cytokines and chemokines. DPP-4 is implicated in the regulation of immune response, viral entry, cellular adhesion, metastasis and chemotaxis. Regarding its numerous substrates and extensive expression inside the body, multitasking DPP-4 has been assumed to participate in different pathophysiological mechanisms. DPP-4 inhibitors or gliptins are increasingly used for the treatment of type 2 diabetes mellitus. Several reports from experimental and clinical studies have clarified that DPP-4 inhibitors exert many beneficial pleiotropic effects beyond glycaemic control, which are mediated by anti-inflammatory, anti-oxidant, anti-fibrotic and anti-apoptotic actions. The present review will highlight the most recent findings in the literature about these pleiotropic effects and the potential mechanisms underlying these benefits, with a specific focus on the potential effectiveness of DPP-4 inhibitors in coronavirus disease-19 and diabetic kidney disease.

Dipeptidyl peptidase-4 (DPP-4) is a ubiquitous, multifunctional, 766-amino acid, type 2 transmembrane glycoprotein, which participates in the regulation of metabolic functions, immune and inflammatory responses, cancer growth and cell adhesion.^1^ It has two forms: the first is a membrane-bound form, which is extensively expressed in the body, including the cells of the immune system, haematopoietic cells, vascular endothelium and epithelial and acinar cells of most tissues; and the second is a soluble circulating form (sDPP-4), which lacks the cytoplasmic and transmembrane domains but retains the catalytic activity and is present in the plasma and other body fluids.^1,2^

As an enzyme, DPP-4 serves as a cell-surface serine ectopeptidase that modifies the bioactivities of several biologically active substrates via cleaving selectively dipeptides from substrates having proline or alanine in the N-terminal penultimate site.^3^ Most notably, DPP-4 has a central role in glucose homeostasis through the degradation of intestinal incretins, mainly glucagon-l ike peptide-1 (GLP-1) and glucose-dependent insulinotropic peptide. Besides intestinal incretins, DPP-4 cleaves and modifies many other substrates, such as cytokines (including interleukin [IL]-3, erythropoietin, granulocyte–macrophage colony-stimulating factor and granulocyte colony-stimulating factor), chemokines (including stromal-derived factor-1 alpha [SDF-1α], monocyte chemo-attractant protein [MCP]-2, granulocyte chemotactic protein-2 and macrophage-derived chemokine) and neuropeptides (including neuropeptide-Y, peptide YY, substance P, endomorphin-2 and bradykinin).^4^

Besides the catalytic activity, DPP-4 also has several non-catalytic functions mediated by its interaction with numerous ligands, such as caveolin-1, collagen, fibronectin, adenosine deaminase, kidney Na^+^/H^+^ ion exchanger-3 (NHE-3), mannose 6-phosphate/insulin-l ike growth factor II receptor, CD-45 and plasminogen 2.^5,6^ For instance, binding of DPP-4 to T cells with adenosine deaminase provides co-stimulatory signals during T-cell activation, with subsequently increased production of interferon-γ and tumour necrosis factor-a lpha (TNF-α).^7^ In addition, DPP-4 expressed on T cells can interact with caveolin-1 on the surface of antigen-presenting cells, leading to its phosphorylation with subsequent induction of downstream nuclear factor-kappa B (NF-κB).^6^ DPP-4 can also regulate cellular adhesion, metastasis and chemotaxis via interaction with extracellular matrix (ECM) proteins, such as collagen and fibronectin.^8^ Interestingly, among its non-catalytic functions, DPP-4 may facilitate the entry of some viruses into host cells.^9^

DPP-4 inhibitors or gliptins are currently approved as anti-hyperglycaemic agents for type 2 diabetes mellitus, with well-proven efficacy and safety. Besides glycaemic control, they offer other benefits, including promoting pancreatic β-cell mass and function, prolonging the satiety time and improving the lipid profile.^10^

Extensive research over the past two decades revealed that the disturbed expression or activity of the multitasking DPP-4 may be involved in various pathological conditions, including inflammatory and immune-mediated and cardiovascular disorders.^11,12^ Accordingly, DPP-4 inhibitors have been demonstrated to possess many pleiotropic actions other than the well-established anti-hyperglycaemic actions, which would enable them to have a decisive role in various disease entities, including neurological, cardiovascular, renal, hepatic and pulmonary diseases (*Table 1*).^11–38^ The current review will highlight the most recent findings about these pleiotropic actions in the literature.

## Anti-inflammatory actions of dipeptidyl peptidase-4 inhibitors

It is obvious that DPP-4 cleaves numerous cytokines, chemokines and peptide hormones implicated in the modulation of immune functions.^39^ In addition, DPP-4 contributes to the maturation of macrophages and dendritic cells, which seems to be independent of its catalytic activity.^6^ Furthermore, DPP-4 has been demonstrated to be involved in antigen-induced T-cell activation by amplifying its signals.^6^ In this respect, treatment of lipopolysaccharide-stimulated murine macrophages with exogenous recombinant DPP-4 resulted in an increase in the expression of toll-l ike receptor (TLR)-4, TLR-2, inducible nitric oxide synthase (iNOS), IL-6, IL-1β and TNF-α.^40^ Consequently, DPP-4 inhibitors are hypothesized as important inflammatory response modulators that could offer a potential therapeutic benefit in inflammatory disease management.^41^

Systemic inflammation may initiate damage to the endothelium, with subsequent infiltration of monocytes, which differentiate into macrophages that secrete several pro-i nflammatory cytokines, resulting in more monocyte recruitment and ultimately vascular damage.^42^ Evidence from experimental studies has referred clearly to the efficacy of DPP-4 inhibitors in attenuating systemic inflammation-induced infiltration of tissue monocytes. In this regard, anagliptin inhibited monocyte–macrophage differentiation and decreased tumour-associated macrophages in non-small-cell lung cancer.^13^ Similarly, alogliptin attenuated the recruitment and chemotaxis of monocytes via GLP-1 receptor (GLP-1R)-dependent downregulation of IL-6 and IL-1β in atherosclerotic lesions in apolipoprotein E-deficient mice.^43^

Inflammation is triggered in many ways, including the upregulation of pro-i nflammatory genes (such as NF-κB, cyclooxygenase and iNOS) along with altered pro-/anti-i nflammatory microRNA (miR) balance.^44^ DPP-4 inhibition has been shown to exert anti-i nflammatory effects by mitigating these inflammatory triggers. Sitagliptin attenuated pro-i nflammatory cytokine production via downregulating the iNOS/nitric oxide/NF-κB pathway in rats with cyclophosphamide-i nduced cerebral toxicity.^14^ Likewise, in lipopolysaccharide-stimulated microglial cells, sitagliptin exerted anti-i nflammatory effects by decreasing the protein levels of pro-i nflammatory cytokines and iNOS.^15^ Vildagliptin attenuated acetic acid-induced colitis by inhibiting the NF-κB signalling and downregulating the pro-i nflammatory miR-146a and inhibited inflammation in the aorta by activating the anti-i nflammatory miR-190a-5p.^45,46^

The transcription factor NF-κB resides in the cytoplasm in an inactive form by binding to its inhibitory subunit, I-κB. Under certain pathological conditions, such as oxidative stress, NF-κB undergoes phosphorylation with subsequent dissociation from its inhibitory subunit and translocation into the nucleus to induce many pro-i nflammatory genes, such as cytokines, chemokines and receptors of advanced glycation end-products (RAGE).^47^ In the same way, sitagliptin has been shown to downregulate NF-κB signalling in the diabetic liver in rats.^16^ Linagliptin attenuated high-methionine-diet-i nduced cardiac hypertrophy in rats by attenuating NF-κB signalling.^17^ Additionally, vildagliptin inhibited carbon tetrachloride-i nduced liver fibrosis and attenuated testosterone-i nduced benign prostatic hyperplasia by targeting NF-κB signalling.^18,48^

TLR-4 is a cell surface pattern-recognizing receptor that has a fate-decisive role in different infections and other human disorders, including malignancy.^49^ Upon binding to a specific ligand, TLR-4 becomes activated to induce a subset of cellular downstream events with subsequent activation of certain transcription factors, including NF-κB, activating protein-1 and interferon regulatory factor, resulting in potent inflammatory responses.^50^ Besides its inflammatory response, TLR-4 plays a crucial role in the initiation and progression of malignant tumours in several ways; it induces the secretion of pro-i nflammatory cytokines and stimulates the recruitment of immune cells, creating a hyperinflammatory state that promotes the secretion of growth, anti-apoptotic and pro-angiogenic factors, as well as ECM-m odifying enzymes that favour the tumourigenesis process.^51^ In other words, TLR-4 can play a decisive role in the development of bacterial infection-i nduced carcinomas, such as gastric, colorectal and lung cancers.^52^ In addition, the development of cancer chemoresistance may be mediated in part via TLR-4; for instance, the reduced TLR-4 expression in macrophages has been linked to glioblastoma-associated immune escape via inhibiting the phagocytic activity of these macrophages.^53^

Given its multiple immunomodulatory and neoplastic actions, the pharmacological modification of TLR-4 signalling may offer a promising therapeutic strategy in cancer management. Of note, DPP-4 and TLR-4 exhibit a crosstalk regulation, as sDPP-4 upregulates TLR-4; meanwhile, TLR-4 induces DPP-4 expression, creating a positive feedback loop that augments the pro-i nflammatory and pro-carcinogenic responses.^40^^,^^54^ Accordingly, the inhibition of DPP-4 has been shown to mitigate inflammation via downregulating TLR-4 signalling.^55,56^ Furthermore, alogliptin, a DPP-4 inhibitor, attenuated diethyl nitrosamine-i nduced hepatocellular carcinoma via TLR-4 downregulation.^57^

**Figure 1:**
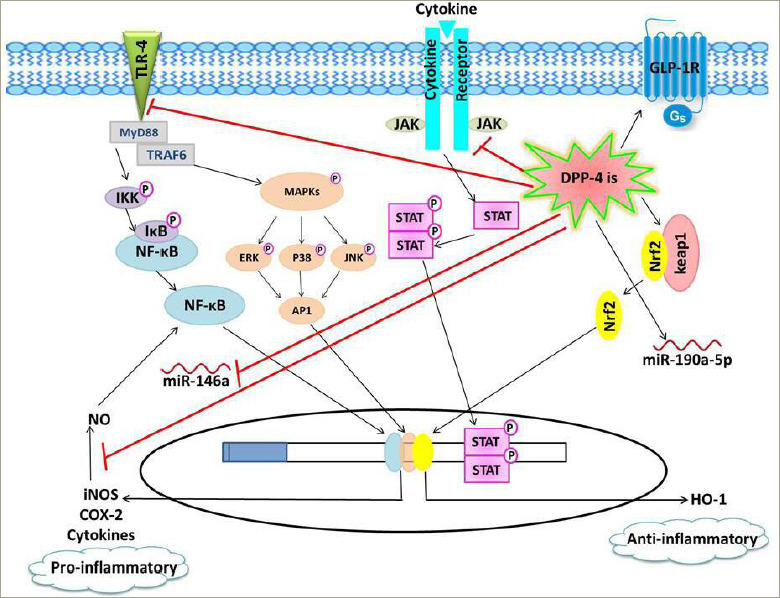
Schematic presentation of the proposed anti-i nflammatory effects of dipeptidyl peptidase-4 inhibitors

In the colon, TLR-4 has an important physiological role in preserving immune homeostasis. However, the overexpression of TLR-4 in the gut tissues promotes inflammation and infiltration of the gut with immune cells, facilitating the initiation and progression of colorectal cancer.^58^ Dysregulation of the gut microbiota may underlie TLR-4 upregulation, resulting in the disturbance in immune homeostasis and ultimately colorectal carcinogenesis.^52^ Interestingly, DPP-4 inhibition could be assumed to prevent microbial dysbiosis-i nduced colorectal TLR-4 upregulation, as vildagliptin has been found to regulate the gut microbiota and prevent the disruption of intestinal immune homeostasis induced by Western diet in mice.^59^ Conclusively, DPP-4 inhibitors could have a tumour suppressor effect on gut carcinogenesis. The proposed anti-i nflammatory effects of DPP-4 inhibitors are summarized in *Figure 1*.

In consonance with the results of experimental studies, DPP-4 inhibitors have been shown to exert anti-i nflammatory effects in humans. A recent meta-analysis of 22 studies enrolling 1,595 patients with type 2 diabetes mellitus revealed that DPP-4 inhibitors exerted anti-i nflammatory effects and reduced the level of IL-6, IL-1β, C-reactive protein and TNF-α.^19^ Another meta-analysis of studies investigating the influence of incretin-based medications in patients with non-alcoholic fatty liver disease revealed clear anti-i nflammatory effects of DPP-4 inhibitors.^60^ Interestingly, the anti-inflammatory effects of DPP-4 inhibitors are not accompanied by severe adverse effects except for a significant correlation with bullous pemphigoid due to altered cytokine expression in skin.^61^

**Table 1:** Summary of the recent clinical and experimental studies on pleiotropic effects of dipeptidyl peptidase-4 inhibitors^13–38^

Intervention	Subjects	Year	Disease/model	Effects	Reference
Sitagliptin	Male Wistar rats	2024	Cyclosporine-induced nephrotoxicity	↑ Nrf-2 ↓ TNF-α, NF-κB and Bax	27
Sitagliptin	Sprague–Dawley rats	2024	5-Flurouracil-i nduced nephrotoxicity	Improved kidney function and structure ↓ TNF-α, IL-1β, NF-κB, Bax:BCL-2 ratio and cleaved capspase-3 ↑ Nrf-2 and renal total anti-oxidant capacity	28
Sitagliptin	Male Wistar rats	2024	Diabetic cardiomyopathy	↓ Integrin-l inked kinase/TGF-β/Smad2/3 ↓Troponin-I , creatine kinase-MB, collagen-I , TNF-α and MMP-9	22
Linagliptin	Mice	2023	Diabetic cardiomyopathy	↓ EndMT, cardiac fibrosis and myocardial fibre and sarcomere disruption ↓ Sox9–necroptosis axis	24
Sitagliptin	Male Wistar rats	2023	Busulfan-i nduced pulmonary and testicular injury	↓ Body weight loss, lung index, MDA, TNF-α and sperm abnormal morphology ↑ Testis index, GSH, testosterone, sperm count, viability and motility Restored SIRT-1/FOXO-1 balance ↓ Collagen deposition and caspase-3	26
Sitagliptin	Male Wistar rats	2023	Streptozotocin-induced type 2 diabetes	↓ PTP-1B/JAK-2/STAT-3 axis	33
Sitagliptin	Male Wistar rats	2023	Streptozotocin-i nduced hepatic injury	↓ Oxidative stress, inflammation, and hepatocyte degeneration ↓ NF-κB, NLRP-3 and mTOR ↑ IKB-α	16
Sitagliptin	Male Wistar rats	2023	Lipopolysaccharide-stimulated microglia	↓ Phagocytic efficiency ↓ iNOS, IL-1β and TNF-α	15
Vildagliptin	Male Wistar rats	2023	Testosterone-i nduced benign prostate hyperplasia	↓ Prostate weight, prostate index, prostate-specific antigen, 5α-reductase activity, dihydrotestosterone ↓ HMGB-1, PI3K/Akt/NF-κB and TNF-α signalling ↑ Nrf-2/HO-1 and GSH levels ↓ MDA and cleaved caspase-3	18
Sitagliptin	Male Wistar rats	2023	Cyclophosphamide neurotoxicity	↑ Nrf-2 ↓ Redox cycle imbalance ↓ iNOS/NO/NF-κB response ↓ Caspase-3/Bax activation	14
Linagliptin	Sprague–Dawley rats	2023	High-methionine-diet-i nduced cardiac hypertrophy	Improved lipid profile, ECG parameters and morphological features of the cardiac muscle ↓ ER stress markers, cardiac markers and NF-κB ↓ Oxidative stress, inflammation and apoptosis	17
Sitagliptin	Human	2023	Type 2 diabetes	↓ lncMIAT, KIM-1 and IL-18	34
Linagliptin	Human	2023	Type 2 diabetes	↓ UACR	32
Sitagliptin	Human	2023	Type 2 diabetes	↓ UACR and systolic blood pressure	36
Vildagliptin	Sprague–Dawley rats	2023	Cisplatin-i nduced chemo-brain	Restored cognitive function and cholinergic neurotransmission ↓ Acetylcholinesterase, neurodegeneration and amyloid plaque deposition ↓ Oxidative stress and neuronal apoptosis ↓ Neuroinflammation ↑ AMPK/Akt/CREB/BDNF	21
Evogliptin	Mice	2023	Diabetic cardiomyopathy	Improved cardiac systolic/diastolic function, hypertrophy and fibrosis ↓ Lipid droplets in the myocardium ↑ Mitochondrial biogenesis	20
DPP-4 inhibitors	Human	2023	Patients with COVID-19 and diabetes	↓ Risk of COVID-19-related death	25
DPP-4 inhibitors	Human	2023	Type 2 diabetes	↓ IL-6, IL-1β, C-reactive protein and TNF-α	19
Linagliptin	Human	2023	HTF culture (HTF)	↓ Fibrosis markers induced by TGF-β ↓ Migration and gel contraction of HTFs ↓ Phosphorylation of Smad2 and Smad3	23
Anagliptin	Mouse	2023	Non-small-cell lung cancer model	↓ Macrophage differentiation and M2 polarization ↓ ROS, NOX1/2 and late ERK signalling	13
DPP-4 inhibitors	Human	2022	Type 2 diabetes	↓ Risk of eGFR decline	31
Gemigliptin	Sprague–Dawley rat	2022	Tacrolimus-induced diabetes	↓ Renal interstitial fibrosis, pro-fibrotic cytokines and oxidative stress ↑ Plasma insulin levels and pancreatic islet	37
Incretin-based therapy	Human	2023	Type 2 diabetes	↓ Albuminuria ↓ Kidney composite outcome	30
Sitagliptin	Mice	2022	Diabetic kidney disease model	Improved kidney pathology ↓ Urinary albumin, creatinine and UACR	29
Sitagliptin	Human	2021	Type 2 diabetes	↓ Urinary albumin excretion ↑ eGFR significantly	38
Linagliptin	Sprague–Dawley rats	2020	Diabetic kidney disease model	↓ Podocyte apoptosis ↑ Insulin/IRS-1/p-Akt signalling ↑ Podocyte Nrf-2 levels	35

*Akt = protein kinase-B; AMPK = adenosine monophosphate-activated protein kinase; Bax = B cell lymphoma 2-associated X; BCL-2 = B cell lymphoma 2; BDNF = brain-derived neurotrophic factor; COVID-19 = coronavirus disease-19; CREB = cAMP-response element-binding protein; DPP-4 = dipeptidyl peptidase-4; ECG = electrocardiogram; eGFR = estimated glomerular filtration rate; EndMT = endothelial-to-mesenchymal transition; ER = endoplasmic reticulum; ERK = extracellular signal-regulated kinase; FOXO-1 = forkhead box protein O-1; GSH = reduced glutathione; HMGB-1 = high-mobility group box-1; HO-1 = haem oxygenase-1; HTF = human Tenon’s fibroblast; IKB-α = nuclear factor-kappa B inhibitor-alpha; IL-1β = interleukin-1 beta; iNOS = inducible nitric oxide synthase; IRS-1 = insulin receptor substrate-1; JAK-2 = Janus kinase-2; KIM-1 = kidney injury molecule-1; lncMIAT = long non-coding myocardial infarction-associated transcript; MDA = malondialdehyde; MMP-9 = matrix metalloproteinase-9; mTOR = mammalian target of rapamycin; NF-κB = nuclear factor-kappa B; NLRP-3 = NLR family pyrin domain-containing-3; NOX = nicotinamide adenine dinucleotide phosphate oxidase; Nrf-2 = nuclear factor erythroid-2-related factor-2; PI3K = phosphatidylinositol-3-kinase; PTP-1B = protein tyrosine phosphatase-1B; ROS = reactive oxygen species; SIRT-1 = sirutin-1; Smad = suppressor of mothers against decapentaplegic; Sox9 = SRY-box transcription factor-9; STAT-3 = signal transducer and activator of transcription-3; TGF-β = transforming growth factor-beta; TNF-α = tumour necrosis factor-alpha; UACR = urinary albumin:creatinine ratio.*

*DPP-4is downregulate pro-inflammatory pathways, including TLR-4/NF-κB, JAK/STAT and iNOS/NO/NF-κB pathways, as well as the pro-inflammatory miR-146a, leading to decreased expression of inflammatory mediators, such as cytokines, COX-2 and iNOS. On the other hand, DPP-4is induce the anti-inflammatory signalling cascades, including GLP-1R/cAMP/protein kinase A and Keap1/Nrf2/HO-1, as well as the anti-inflammatory miR-190a-5p.*

*AP-1 = activator protein1; cAMP = cyclic adenosine monophosphate; COX-2 = cyclooxygenase-2; DPP-4 = dipeptidyl peptidase-4; DPP-4is = dipeptidyl peptidase-4 inhibitors; ERK = extracellular signal-regulated kinase; GLP-1R = glucagon-like peptide-1 receptor; Gs = stimulatory G protein; HO-1 = haem oxygenase-1; IKB = inhibitor of NF-κclB; IKK = inhibitor of NF-κB kinase; iNOS = inducible nitric oxide synthase; JAK = Janus kinase;JNK = c-Jun N-terminal kinase; Keap-1 = Kelch-like ECH-associated protein 1; MAPKs = mitogen-activated protein kinases; miR = microRNA; MyD88 = myeloid differentiation primary response 88; NF-κB = nuclear factor-κ beta; NO = nitric oxide; Nrf-2 = nuclear factor erythroid-2-related factor; STAT = signal transducer and activator of transcription; TLR-4 = toll-like recptor-4; TRAF6 = tumour necrosis factor receptor-associated factor 6.*

## Anti-oxidant effects of dipeptidyl peptidase-4 inhibitors

Free radicals are active molecules generated physiologically during various biological processes and participate in physiological reactions. However, the excess free radicals can interact with many biologically important molecules, interfering with their physiological functions.^62^ Moreover, they are potent upstream regulators of many pathological pathways, such as apoptosis, necrosis and inflammation. On the other hand, the intrinsic anti-oxidant defence system, which has enzymatic and non-enzymatic components, neutralizes the excessive amounts of free radicals and minimizes their harmful impact.^62^ A pathological state of oxidative stress ensues when free radicals exceed the neutralizing capacity of the anti-oxidant system. Hereafter, the anticipation of oxidative stress and the restoration of the physiological oxidant/anti-oxidant balance are pivotal to precluding the initiation and progression of many disease states.^62^

Several recent reports from experimental studies have clarified the anti-oxidant effects of DPP-4 inhibitors in different tissues.^20,63–65^ Mounting preclinical evidence proposes that pharmacological or genetic inhibition of DPP-4 can restore the oxidant/anti-oxidant balance and improve the lipid metabolism by supporting the intrinsic anti-oxidant defence system while inhibiting the expression/activity of pro-oxidant enzymes.^65–67^ For instance, genetic DPP-4 deficiency attenuated oxidative stress in an experimental diabetic nephropathy model.^66^ DPP-4 inhibitors decreased the hypoxia-induced upregulation of the pro-oxidant enzyme nicotinamide adenine dinucleotide phosphate oxidase (NOX) in rat cardiomyocytes.^68^ Saxagliptin prevented NOX-mediated endothelial nitric oxide synthase uncoupling and attenuated vascular remodelling in diabetic mice.^69^ Vildagliptin restored the oxidant/anti-oxidant balance by increasing the superoxide dismutase and glutathione content in rats with testosterone-induced benign prostatic hyperplasia and cisplatin-induced neurotoxicity.^18,21^

Mitochondrial dysfunction is an important source of oxidative stress and represents a causative factor in several diseases.^70^ The inhibition of DPP-4 was demonstrated to mitigate oxidative stress by endorsing mitochondrial function; evogliptin mitigated mitochondrial free radical production and endorsed mitochondrial biogenesis in a diabetic cardiomyopathy mouse model.^20^ In addition, saxagliptin moderated hypoxia-i nduced damage in rat cardiomyocytes by rescuing mitochondrial membrane potential.^68^

The production of inflammation-i nduced reactive oxygen species (ROS) increases the progression of several pathological conditions in a reciprocal manner.^71^ In this context, DPP-4 inhibitors mitigated inflammation-i nduced oxidative stress in GLP-1R-dependent and GLP-1R-i ndependent manners.^63,64,72^

Advanced glycation end-products (AGEs), generated by the non-enzymatic Maillard reaction due to their exposure to saccharides, interact with RAGE to endorse oxidative stress.^73^ Moreover, the AGE/RAGE axis forms a positive feedback loop with DPP-4 to intensify oxidative stress, as AGE-/RAGE-generated ROS induces the release of DPP-4 from the endothelial cells, which upregulates RAGE to additionally augment AGE actions.^74^ Therefore, DPP-4 inhibitors could mitigate oxidative stress by mutation of the crosstalk between the AGE/RAGE axis and DPP-4. Accordingly, sitagliptin inhibited arterial calcification by downregulating RAGE in mice.^63^ Saxagliptin mitigated isoproterenol-i nduced myocardial injury by inhibiting AGE/RAGE signalling in diabetic rats.^75^ In addition, linagliptin attenuated the positive feedback loop between AGE/RAGE axis and DPP-4 in human umbilical vein endothelial cells.^76^

Interestingly, several clinical reports suggest a promising role of incretin-based medications, including DPP-4 inhibitors, against oxidative stress in humans.^77–79^ Conversely, some randomized controlled trials could not prove any beneficial effect of DPP-4 inhibitors on oxidative milieu, warranting further clinical investigations to elucidate the ultimate efficacy of DPP-4 inhibitors in alleviating oxidative stress in humans.^80–82^

## Anti-fibrotic effects of dipeptidyl peptidase-4 inhibitors

Fibrotic disorders encompass a wide range of clinical entities involving a sophisticated and multistage course of tissue damage and inflammation. ECM expansion constitutes the pathological landmark of fibrosis. It is regulated by a series of cytokines, chemokines, growth factors, adhesion molecules and signalling transduction processes.^83^

Growing evidence proposes that DPP-4 is a pro-fibrotic agent with a central role in fibrogenesis in different organs. DPP-4 inhibitors have been demonstrated to exert their anti-fibrotic actions in various organs, such as liver, lungs, skin, heart, kidneys and eyes.^22,23,84–87^

Fibroblasts constitute the primary ECM-secretory cells. Under normal physiological conditions, fibroblasts preserve the matrix network through basal ECM deposition/degradation. However, under stressful circumstances, they become differentiated into myofibroblasts.^88^ Myofibroblasts have an increased capacity to secrete ECM proteins, ultimately converting healthy tissues into non-functional fibrotic tissues. Besides ECM production, myofibroblasts secrete proliferative mediators, such as vascular endothelial growth factor-A, transforming growth factor-beta (TGF-β) and pro-i nflammatory factors such as IL-1, IL-6, IL-8 and MCP-1.^88,89^

Fibroblasts exhibiting increased myofibroblast markers are isolated from patients with systemic sclerosis and have extensive DPP-4 expression.^86^ In addition, DPP-4-expressing fibroblasts were found to constitute the majority of connective tissues, which were deposited in the skin after surgical wounding.^90^ Consistently, DPP-4 inhibitors decreased scarring without adversely affecting wound healing, highlighting the selective expression pattern of DPP-4 in fibroblast population with a high capability of ECM production, rather than those with more homeostatic functions.^90^

Endothelial-to-mesenchymal transition (EndMT) is another source of myofibroblasts. Cells of the endothelial layer detach, lose all endothelial markers and attain mesenchymal phenotype in a sophisticated process. They transform into myofibroblastic cells, which infiltrate the interstitial tissues to induce fibrosis by excessive secretion of collagen, α-smooth muscle actin and other ECM proteins.^91^

It has been demonstrated that DPP-4 promotes EndMT; thus, DPP-4 inhibitors may possibly exert anti-fibrotic effects through the regulation of EndMT.^92^ In this context, linagliptin ameliorated cardiac fibrosis in diabetic mice through suppression of EndMT.^24^ Integrins, which act as cell surface receptors, promote EndMT and fibrosis through interaction with the membrane-bound DPP-4.^92^ The abrogation of DPP-4/integrin interactions would have an anti-fibrotic effect through suppression of EndMT. Linagliptin mitigated EndMT by inhibiting these interactions between DPP-4 and integrin-β1.^92^ The key anti-fibrotic miR-29 can also interrupt the DPP-4/integrin interactions through negative regulation of DPP-4 gene expression. The miR-29 also downregulates the ECM genes, protecting different organs from EndMT and fibrotic damage.^93^ In this regard, the suppressed miR-29 expression in diabetic mouse kidneys was associated with the upregulation of DPP-4 and EndMT, while linagliptin administration attenuated EndMT through restoration of normal miR-29 expression.^93^

TGF-β ligands are a superfamily of cytokines recruited in the physiology of proliferation, differentiation, migration and immunity. Under certain pathological triggers, TGF-β, ‘the master regulator of fibrosis’, can activate fibroblast and induce EndMT.^94^ TGF-β is implicated in the fibrotic process through canonical and non-canonical signalling pathways: the canonical TGF-β signalling involves the phosphorylation of suppressor of mothers against decapentaplegic (Smads), specifically the receptor-associated Smads (Smad 1/2/3). Targeting the Smad signalling pathway can offer a promising fibrosis treatment strategy.^94^ In addition, pharmacological DPP-4 inhibition can suppress TGF-β2-induced EndMT in cultured human dermal microvascular endothelial cells through suppression of Smad3 phosphorylation.^93^

Moreover, complete TGF-β response requires non-canonical signalling cascades comprising different mitogen-activated protein kinases (MAPKs), namely, the extracellular signal-regulated kinase (ERK), c-Jun N-terminal kinase (JNK) and phosphatidylinositol-3-kinase/protein kinase-b (Akt), Rho-l ike GTPases (Rho) and TNF receptor-associated factor 4/6 pathways.^94^ ERK is of particular importance among non-canonical TGF-β signalling molecules. Sitagliptin attenuated hepatic fibrosis in diet-i nduced non-alcoholic fatty liver models in rats by normalizing ERK signalling.^84^ In their study, Hong et al. stated that gemigliptin attenuated both TGF-β canonical and non-canonical pathways; it inhibited EndMT in human umbilical vein endothelial cells by suppression of Smads, ERK and JNK.^95^

Both DPP-4 and TGF-β presumably exhibit a pro-fibrotic crosstalk regulation, as membrane-bound DPP-4 is critical for TGF-β-induced receptor heterodimerization and consequent intracellular downstream signalling that ultimately triggers EndMT.^96^ Simultaneously, TGF-β triggers the upregulation of membrane-bound DPP-4, followed by downstream signalling.^97^ Accordingly, the activation of cultured normal human dermal fibroblasts with TGF-β increased the protein levels of membrane-bound DPP-4 via ERK signalling.^86^ Mitigation of the DPP-4/TGF-β crosstalk is assumed to contribute to the anti-fibrotic actions of DPP-4 inhibitors. The inhibition of DPP-4 by linagliptin mitigated fibrosis following glaucoma filtering surgery by inhibiting the TGF-β-mediated Smad2/3 phosphorylation.^23^ Sitagliptin exerted cardioprotective effects through attenuation of the TGF-β/Smad signalling pathway in a rat model of diabetic cardiomyopathy.^22^ It attenuated *in vitro* fibroblast activation in lung culture by decreasing TGF-β-mediated Smad3 phosphorylation.^85^ In the same way, vildagliptin ameliorated renal injury after hepatic ischaemia/reperfusion injury through TGF-β/Smad downregulation.^98^ Considering the effect on non-canonical pathways, the pharmacological DPP-4 inhibition has been revealed to repress fibroblast activation in cultured human dermal fibroblasts through attenuation of the TGF-β-induced activation of ERK signalling.^86^ Furthermore, alogliptin alleviated cyclophosphamide-i nduced kidney injury in rats by suppressing TGF-β-induced phosphorylation of Smad3 and JNK.^87^

An additional role of DPP-4 in fibrosis is mediated by sDPP-4, which has been shown to induce fibrosis-associated proteins in primary human dermal fibroblasts, suggesting that sDPP-4 is not just an activation marker but is functionally needed for activation of fibroblast and progression of fibrosis.^83^ The pro-fibrotic signalling of sDPP-4 comprises phosphorylation of NF-κB and Smad pathways independent of TGF-β, while TGF-β activates Smad, ERK and NF-κB downstream signalling by binding to TGF-β receptors, and sDPP-4 activates NF-κB and Smad signalling via proteinase-activated receptor-2 (PAR-2), which acts as a receptor for sDPP-4.^83^ The inhibition of DPP-4 may result in abrogation of sDPP-4/PAR-2 interactions, thus exerting anti-fibrotic effects. This was illustrated by a study with linagliptin, which prevented sDPP-4 interactions with ECM components, receptors or plasma membrane components, attenuating ECM and intracellular signal transduction.^99^

## Potential anti-inflammatory and anti-fibrotic effects of dipeptidyl peptidase-4 inhibitors in coronavirus disease-19

The coronavirus disease-19 (COVID-19) pandemic, caused by severe acute respiratory syndrome coronavirus 2 (SARS-CoV-2), has been threatening global public health since December 2019. The end of the pandemic is far unattainable, as the total confirmed cases and deaths continue to increase rapidly.^3^

The potential effects of various anti-hyperglycaemic drugs on the prognosis of COVID-19 have raised an increasing interest due to the association between diabetes mellitus and the possibility of developing more severe forms of the disease.^100^

Among different anti-diabetic drugs, DPP-4 inhibitors have a special importance because of their potential contribution to inflammatory and immune responses.^1^ The interaction between the surface antigen spike glycoprotein of SARS-CoV-2 and TLR-4 brings inflammatory cascades that ultimately lead to the development of cytokine storm and subsequently multi-organ failure.^101^ Conversely, DPP-4 inhibition has been shown to attenuate TLR-4 activation in lung tissues, suggesting that DPP-4 inhibitors could abrogate the sequences of these SARS-CoV-2/TLR-4 interactions.^102^

Interestingly, the identification of the membrane-bound DPP-4 as a functional receptor for Middle East respiratory syndrome coronavirus (MERS-CoV) proposes that it may serve as a potential receptor for the spike protein of the genetically similar SARS-CoV-2.^9^ Although evidence refers to the membrane-bound angiotensin-converting enzyme-2 but not DPP-4 as the major functional receptor protein for SARS-CoV-2, the finding that SARS-CoV-2 interacts with sDPP-4 in the sera of patients with convalescent COVID-19 proposes a modulatory role for DPP-4 in the development of COVID-19 infection.^103^ Considering the distribution pattern of DPP-4 in the human respiratory tract, it can promote the development of cytokine storms and fatal COVID-19 outcomes by facilitating viral entry into the respiratory tract. Accordingly, the use of DPP-4 inhibitors in patients with COVID-19, either diabetic or not, can simply decrease the entry and replication of SARS-CoV-2 in the lungs.^104^

A population-based study clarified that the use of DPP-4 inhibitors in patients with diabetes reduced severe symptoms of COVID-19 by 64% compared with non-users.^105^ Another case–control retrospective study that precisely assessed sitagliptin administration and recruited a relatively large number of patients reported a significant decrease in intensive care unit (ICU) admission and mortality in the sitagliptin group versus non-users.^106^ More recently, an updated living systematic review and meta-analysis demonstrated that patients with a more severe course of diabetes had a worse prognosis of COVID-19 compared with patients with less severe diabetes. The same study reported with high certainty evidence of a decreased risk of COVID-19-related death with the use of DPP-4 inhibitors.^25^

Notably, patients with COVID-19 who develop pneumonia often progress rapidly into pulmonary fibrotic changes. The extreme lung damage caused by these fibrotic processes can negatively impact the functional capacity and life quality among COVID-19 survivors.^107^ In addition, critically ill COVID-19 cases admitted to ICU commonly develop multi-organ damage.^108^ This might be of special importance for patients with diabetes with impaired lung, heart and kidney functions, making them predominantly vulnerable to cumulative injury during SARS-CoV-2 infection. Therefore, the administration of DPP-4 inhibitors to patients with diabetes and COVID-19 could have a meaningful role in the prevention of long-term complications of COVID-19 through their well-i dentified anti-fibrotic, anti-i nflammatory, cardioprotective and nephroprotective actions.^11,12^

These pleiotropic anti-i nflammatory and anti-fibrotic actions of DPP-4 inhibitors are not analogous to those of the standard anti-i nflammatory agents (such as corticosteroids) and may provide a helpful tool in COVID-19 therapy owing to their other positive actions.^109^

## Anti-apoptotic effects of dipeptidyl peptidase-4 inhibitors

Apoptosis is a programmed cell death intended to remove harmful cells, such as those with malignant mutations or DNA damage. It comprises a series of strictly regulated events that ultimately result in the clearance of the damaged cells without initiating an inflammatory response. Meanwhile, dysregulation of apoptosis is indulged in the development of numerous inflammatory, malignant and degenerative diseases.^110^

Dozens of experimental studies have clarified the important anti-apoptotic effects produced by DPP-4 inhibition in various organs. For example, vildagliptin exerted an anti-apoptotic effect in cisplatin-i nduced chemo-brain in rats.^21^ Sitagliptin inhibited busulfan-i nduced pulmonary and testicular apoptosis in rats.^26^ It also prevented apoptosis in diabetic rat testes.^111^ Omarigliptin mitigated rotenone-i nduced parkinsonism in rats via its anti-apoptotic effects.^112^

Inhibitors of DPP-4 exerted anti-apoptotic effects mainly through GLP-1R-dependent mechanisms. The cyclic adenosine monophosphate (cAMP)/protein kinase-A (PKA) signalling pathway underlies the GLP-1R-mediated anti-apoptotic effects.^113^ GLP-1R brings PKA-dependent phosphorylation of the transcription factor cAMP-responsive element-binding protein that promotes cell survival.^114^ In this regard, sitagliptin promoted functional recovery and axonal regeneration following spinal cord injury in the rat through GLP-1R-mediated anti-apoptosis.^115^ The topical application of GLP-1R agonist precluded retinal apoptosis in spontaneous diabetic mice.^116^ Similarly, stimulation of the cardioprotective signalling by GLP-1 has been demonstrated to inhibit cardiomyocyte apoptosis.^117^

Numerous internal and external stimuli, including oxidative stress, DNA mutations and viral infection, trigger a series of intracellular signalling pathways to initiate apoptosis. These pathways involve the activation of caspases, which are proteolytic enzymes that play a crucial role in apoptosis.^118^ Caspases are inactive pro-enzymes that undergo cascade proteolytic cleavage upon activation by intrinsic or extrinsic stimuli. The activated caspase-8 and caspase-9 cleave and activate effector caspases, such as caspase-3 and caspase-7, which consecutively induce DNA fragmentation and apoptosis.^118^ Vildagliptin exerted anti-apoptotic effects by downregulating caspase-3 expression in cisplatin-i nduced neurotoxicity, manganese-i nduced nephrotoxicity and hepatic ischaemia/reperfusion injury.^21,119,120^ Sitagliptin inhibited busulfan-i nduced apoptosis in pulmonary and testicular tissues, as well as cyclophosphamide-i nduced cerebral neuronal apoptosis by downregulating the caspase-3 expression.^14,26^ In diabetic rat testes, sitagliptin inhibited caspase-3 and caspase-12.^111^ Similarly, omarigliptin reduced endoplasmic reticulum pro-apoptotic caspase-12 in rotenone-treated rats.^112^

Oxidative stress is an important intrinsic trigger of apoptosis. It causes the release of the mitochondrial cytochrome-c into the cytosol, resulting in the activation of caspase-9 to initiate the caspase cascade.^121^ Recent experimental studies have clarified that DPP-4 inhibitors could alleviate the oxidative stress-i nduced apoptosis.^14,21,26,112^

On the other hand, pro-i nflammatory cytokines, such as TNF-α and IL-1, represent an extrinsic pathway that triggers apoptosis by binding to their cognate receptors with subsequent activation of caspase-8.^110^ Linagliptin suppressed apoptosis of retinal capillary cells in experimental diabetic retinopathy through inhibition of IL-1 -mediated inflammatory response.^122^ It produced a neuroprotective effect in hyperglycaemic mice with stroke through anti-apoptotic and anti-i nflammatory mechanisms.^123^ Furthermore, gemigliptin exerted anti-apoptotic and anti-i nflammatory effects in a murine model of adriamycin-i nduced nephropathy.^124^ Sitagliptin prevented apoptotic cell death and downregulated the pro-inflammatory cytokines, TNF-α and IL-1β, in type 2 diabetic rats.^125^

The B cell lymphoma-2 (BCL-2) family of proteins has a central role in modulating the intrinsic pathway of apoptosis through controlling mitochondrial permeability. The BCL-2 protein family comprises two classes: anti-apoptotic BCL-2 proteins (such as BCL-w, BCL-2 and BCL-xL) and pro-apoptotic BCL-2 proteins (such as BAX, Bak, Bok and BAD).^110^ The relative pro-apoptotic/anti-apoptotic gene expressions regulate the cellular response to apoptotic signals. Thus, a higher BCL-2:BAX ratio is essential for cell survival, while the opposite triggers apoptosis.^126^

Experimental research clearly denoted that inhibition of DPP-4 has inhibited apoptosis in several models through restoration of the anti-apoptotic:pro-apoptotic BCL-2 ratio. Consistently, vildagliptin increased BCL-2 expression and downregulated BAX and caspase-3 expression in Alzheimer’s disease model and cisplatin-induced hippocampal neuronal toxicity model.^21,127^ Sitagliptin counteracted the increase in the BAX:BCL-2 ratio in the kidney of type 2 diabetic rats.^125^ Similarly, it inhibited the alteration of BAX activation in the brain of cyclophosphamide-treated rats.^14^ Gemigliptin upregulated BCL-2 and diminished the BAX:BCL-2 ratio as well as the cleavage of caspase-3, caspase-8 and caspase-9 in the heart of diabetic mice.^128^

Other intracellular molecular mechanisms have been suggested from experimental lessons to be implicated in the beneficial anti-apoptotic actions of DPP-4 inhibitors. DPP-4 inhibitors may produce anti-apoptotic effects by modulating Rho-A, which regulates the cell behaviour and cytoskeletal dynamics.^129^ The decreased expression of Rho-A has been linked to increased apoptosis.^130^ DPP-4 activity has been found to enhance apoptosis through the downregulation of Rho-A, with subsequent destruction of the podocyte cytoskeleton.^131^ Contrariwise, the inhibition of DPP-4 used cellular protection through restoration of the normal Rho-A level.^131,132^ The inhibition of DPP-4 by gemigliptin produced anti-apoptotic and anti-angiogenic actions in the retinas of spontaneous diabetic mice and ischaemia-i nduced retinopathy mice by decreasing the expression of plasminogen activator inhibitor-1 (PAI-1).^133^ Another study revealed that vildagliptin has suppressed apoptosis in diabetic rats through inhibition of miR-375-3p, which in turn activated 3-phosphoinositide-dependent protein kinase-1.^45^

Exceptionally, one study showed that sitagliptin exerted pro-apoptotic effects in human pulmonary arterial smooth muscle cells via upregulating the phosphatase and tensin homologue deleted on chromosome 10/Akt/p38MAPK/ERK1/2 signalling pathway.^134^ However, this unusual result does not exclude the obvious anti-apoptotic outcomes of DPP-4 inhibitors obtained in the majority of experimental studies.

## Potential pleiotropic effects of dipeptidyl peptidase-4 inhibitors in diabetic kidney disease

Diabetic kidney disease (DKD) is the leading cause of renal failure, necessitating renal replacement therapy globally. Despite extensive research on the underlying pathophysiological roots of DKD, the available therapies failed to lower its prevalence over the past three decades.^135^

In a recently published narrative review of the evidence-based therapies of DKD, DPP-4 inhibitors have been addressed as one of the four effective therapeutic approaches.^136^ In accordance with this, one study suggested DPP-4 inhibitors as one of the albuminuria-l owering agents, which can be effectively used by crossover rotation to overcome resistance to renin–angiotensin–aldosterone system (RAAS) inhibitors.^137^

The renal distribution of DPP-4 involves the proximal tubular brush border, Henle’s loop, distal and collecting ducts and glomerular epithelial and endothelial cells.^138^ The increased expression/activity of DPP-4 has been linked to the onset and progression of DKD.^139–141^ The upregulation of DPP-4 in diabetic glomeruli could have a role in DKD pathogenesis in several ways; DPP-4 can reduce the natriuretic and diuretic effects of GLP-1 in the kidney.^142^ DPP-4-i nduced inactivation of SDF-1α could exaggerate hypoxia-i nduced podocyte loss.^142^ The interaction between DPP-4 and ECM proteins, such as integrin-β1, promotes EndMT by inducing vascular endothelial growth factor receptor-1 (VEGFR-1) in endothelial cells.^92^ The membrane-bound DPP-4 can also promote EndMT via activating the cation-independent mannose 6-phosphate receptor to stimulate the TGF-β/Smad signalling pathway.^76^ Moreover, sDPP-4 released from endothelial cells as a result of AGE/RAGE interaction can activate mannose 6-phosphate receptors to further stimulate AGE/RAGE signalling in a reciprocal manner.^74^ Finally, DPP-4 can modulate the immune and inflammatory responses in the diabetic kidney through its effects on different inflammatory cells and mediators.

Several experimental studies have clarified the positive effects of DPP-4 inhibition on renal pathogenic processes, including oxidative stress, inflammation, natriuresis, apoptosis, albuminuria and fibrosis under diabetic and non-d iabetic conditions.^27–29^ More precisely, the experimental studies conducted by Mima et al. have found that DPP-4 inhibitors exerted renoprotective effects primarily on the podocytes and the endothelial cells rather than on the mesangial cells. This finding has been confirmed by many large-scale clinical trials, including The Cardiovascular and Renal Microvascular Outcome Study with Linagliptin in Patients With Type 2 Diabetes Mellitus (CARMELINA; ClinicalTrials.gov identifier: NCT01897532), which clarified that DPP-4 inhibitors could mitigate albuminuria, although they failed to improve composite renal outcomes significantly.^30,143–145^ However, a retrospective study on the effect of DPP-4 inhibitors in patients with type 2 diabetes found that DPP-4 inhibitors were correlated with lower risks of a decline in the estimated glomerular filtration rate.^31^ Conversely, a recent small-scale clinical trial for the evaluation of the effect of linagliptin on microalbuminuria in type 2 diabetes patients with nephropathy (IRCT20171030037093N11) found no significant difference in albuminuria between linagliptin and placebo.^32^ These discrepant results may be attributed to shorter periods of follow-up or a smaller number of enrolled patients.

Hyperglycaemia is the most proximal provocative factor implicated in the initiation and progression of DKD. It is well known that DPP-4 inhibitors can effectively neutralize this important aetiological factor and achieve euglycaemia in a GLP-1R-dependent manner. However, a recent study showed that DPP-4 inhibitors failed to decrease the progression of kidney damage despite reducing hyperglycaemia and renal DPP-4 activity in a murine model of DKD, suggesting that controlling hyperglycaemia alone is not sufficient for DKD prevention.^146^

The pathogenesis of DKD also involves the activation of a plethora of potential biochemical pathways including but not limited to the activation of diacylglycerol (DAG)/protein kinase Cβ (PKCβ) and AGE/RAGE axes and RAAS, oxidative stress, inflammation, albuminuria, EndMT and glomerular hyperfiltration.^147^ Besides their anti-hyperglycaemic effects, DPP-4 inhibitors could exert renoprotective effects in DKD through pleiotropic actions that are mediated via GLP-1R-dependent and GLP-1R-independent mechanisms.^136^

The renal expression of GLP-1R, which is mainly confined to glomerular tissues, is downregulated in long-standing type 1 diabetes.^148,149^ In the setting of hyperglycaemia, the increased PKCβ signalling abolishes the renal beneficial GLP-1R-mediated effects via ubiquitination and downregulation of GLP-1R in the glomerular tissues.^148,150^ The activation of DAG/PKCβ signalling can also contribute to DKD development through the induction of ECM accumulation, podocyte apoptosis and inflammation.^151^ In addition, PKCβ acts in a reciprocal way to increase oxidative stress, as it activates mitochondrial NOX to induce ROS generation; meanwhile, ROS and AGEs increase DAG levels to stimulate PKCβ.^152^^,^^153^ Several experimental studies have demonstrated that DPP-4 inhibitors can inhibit PKCβ phosphorylation/signalling in GLP-1R-dependent and GLP-1R-independent mechanisms, abolishing its injurious effects on the diabetic kidney.^154–156^

The upregulation of angiotensin II caused by RAAS activation in the kidney exerts deleterious pro-fibrotic effects via induction of p-ERK-1/2/PAI-1 signalling. The GLP-1R agonism has been shown to reverse these angiotensin II-i nduced pro-fibrotic renal effects via activation of the cAMP/PKA pathway.^149^ Similarly, GLP-1R/cAMP signalling attenuated inflammation and oxidative stress elicited by the AGE/RAGE axis in mesangial cells, downregulated pro-i nflammatory markers (CD-68 and chemokine [C-X-C motif] ligand-2) in the cortex of diabetic mice kidney and reduced microalbuminuria and mesangial expansion via inhibiting TGF-β.^148,149,157^ Other protective effects exerted by GLP-1R in DKD involve diuretic effects mediated by the inhibition of NHE-3 directly and sodium/glucose cotransporter-2 indirectly, as well as the suppression of the sympathetic overactivity.^149,158^

Inflammation and oxidative stress associated with cellular glucotoxicity are crucial mechanisms in DKD pathogenesis.^159^ The hyperglycaemic state stimulates macrophages and T cells to secrete pro-i nflammatory cytokines.^157^ NF-κB activation mediated by hyperglycaemia increases the transcription of several cytokines, chemokines and adhesion molecules, such as TNF-α, IL-6, MCP-1, vascular cell adhesion molecule-1 and intercellular adhesion molecule-1.^160,161^ In this context, sitagliptin attenuated diabetic nephropathy in rats through anti-inflammatory effects mediated by the downregulation of the protein tyrosine phosphatase-1B/Janus kinase-2/signal transducer activator of transcription-3 axis.^33^ It protected against DKD in patients with type 2 diabetes via downregulating long non-coding myocardial infarction-associated transcript and decreasing the levels of kidney injury markers and pro-i nflammatory cytokines.^34^ In addition, DPP-4 inhibitors exerted potent anti-i nflammatory effects through activation of the nuclear factor erythroid-2-related factor-2 (Nrf-2)/haem oxygenase-1 pathway and downregulation of the TLR-4/NF-κB pathway.^55,162^ Furthermore, sitagliptin exerted anti-oxidant, anti-i nflammatory and anti-apoptotic effects in experimental cyclosporine-i nduced nephrotoxicity via upregulation of Nrf-2 and suppression of TNF-α, NF-κB and Bax.^27^ DPP-4 inhibitors exerted anti-oxidant effects by downregulating miR-200a that inhibits the Nrf-2-/Kelch-l ike epichlorohydrin (ECH)-associated protein-1 pathway.^163^ Nrf-2 is an important component of the intrinsic antioxidant system that regulates cellular responses to stress and maintains redox homeostasis.^164^

Podocyte apoptosis has a central role in the development of albuminuria in DKD. Proper insulin signalling is essential for podocyte differentiation and survival.^35,165^ The increased activity of glomerular PKCβ has been linked to insulin receptor substrate-1 (IRS-1) dysfunction and insulin resistance in diabetic rats.^150^ The inhibition of glomerular insulin signalling can induce podocyte apoptosis via altering VEGFR activity, which is further altered by the induction of the Src homology-2 domain-containing phosphatase-1 in DKD.^166^ Outstandingly, Mima et al. showed that linagliptin improved renal pathology and function in experimental DKD through restoration of normal glomerular insulin signalling and activation of insulin/IRS-1/p-Akt signalling, which was mediated in part by increasing podocyte Nrf-2 levels.

The increased expression of DPP-4 in podocytes may add to podocyte loss via SDF-1α degradation.^131^ The inhibition of DPP-4 by linagliptin prevented the effacement of podocyte foot process and proteinuria by increasing endothelial SDF-1α in Zucker obese rats.^167^ It ameliorated albuminuria and podocyte loss in the kidney of GLP-1R-deficient diabetic mice in an SDF-1α-dependent manner.^168^ Of note, saxagliptin inhibited podocyte loss via preserving synaptopodin and Rho-A.^131^

EndMT is a key process in the development of renal sclerosis in DKD.^93,169^ Oxidative stress and inflammation as well as RAAS activation associated with hyperglycaemia induce TGF-β expression and ECM deposition, leading ultimately to glomerulosclerosis via NF-κB activation.^170^ Of note, downregulation of the anti-fibrotic miR-29 has been linked to EndMT in experimental DKD.^93^ In this context, linagliptin attenuated renal fibrosis through the upregulation of miR-29.^93^

Glomerular injury induced by hyperfiltration and increased glomerular capillary pressure is important for DKD pathogenesis. Hypertension, glycosuria and hyperglycaemia-i nduced upregulation of NHE-3 in the renal proximal tubules all contribute to glomerular hyperfiltration.^135^ DPP-4 inhibitors are assumed to modulate the contributing mechanisms involved in glomerular hyperfiltration in a GLP-1R-dependent manner. GLP-1R activation reduces hyperglycaemia and mediates vascular relaxation and NHE-3 inactivation, leading to decreased proximal sodium reabsorption and natriuresis, thus protecting against hypertension and glomerular hyperfiltration.^149,171^

## Conclusion

The heavy expression pattern, as well as the plentiful substrates of the multifunctioning DPP-4, nominates it to be indulged in various pathophysiological processes in almost all body tissues via modulating different intracellular molecular pathways that mediate these processes. Hence, the current article has focused on the several beneficial pleiotropic effects produced by the pharmacological inhibition of DPP-4 by gliptins in several experimental models and its potential clinical implications in human diseases.
